# Antibody-based intervention against the pore-forming toxins of *Staphylococcus* *aureus*

**DOI:** 10.1080/21505594.2017.1421831

**Published:** 2018-03-19

**Authors:** Isaac P. Thomsen

**Affiliations:** Department of Pediatrics, Division of Pediatric Infectious Diseases and the Vanderbilt Vaccine Research Program, Vanderbilt University Medical Center, Nashville, Tennessee, USA

**Keywords:** antibody, leukocidin, *Staphylococcus aureus*, toxin

*Staphylococcus aureus* is nearly unrivaled among bacterial pathogens, both in the diversity of clinical syndromes it causes (from skin abscesses and food-borne illness to endocarditis and sepsis) and in its complex array of virulence mechanisms. In addition, antimicrobial resistance rates continue to increase, both in methicillin-susceptible (MSSA) and methicillin-resistant (MRSA) isolates [1]. *S. aureus* harbors numerous gene regulatory and quorum sensing systems, and features substantial genomic plasticity and frequent redundancy among specific virulence factors. For these reasons, this pathogen represents a daunting target for the development of novel therapeutic and preventive measures, dating back to the first published attempt at a *S. aureus* vaccine in 1902 [2].

Since the onset of the community-associated MRSA epidemic, a family of toxins produced by *S. aureus*, the leukocidins, have gained increasing attention as important virulence determinants and potential targets of intervention against this pathogen. The leukocidins known to be produced by clinical isolates of *S. aureus* include Panton-Valentine leukocidin (PVL), LukAB (also known as LukGH), LukED, and the γ-hemolysins HlgAB and HlgCB [3]. Each of these two-component toxins is secreted as a pair of monomers that oligomerize to form a pore on the surface of phagocytes, lymphocytes, and natural killer cells, and they are important mediators of staphylococcal evasion of innate host defenses. The neutrophil represents the primary innate defense against *S. aureus* infection in humans, as evidenced in part by the clear predilection toward invasive *S. aureus* disease in patients with neutrophil defects [4]. The leukocidins exert their effect at the level of the neutrophil and other phagocytes, binding receptors in the chemokine and complement receptor families [5–8], forming a pore, and potently lysing these cells, thereby facilitating infection in a variety of *in vivo* models [9–13]. *S. aureus* elaborates a number of additional pore-forming toxins outside the leukocidin family, prominently including alpha-hemolysin (Hla), which primarily targets erythrocytes, epithelial and endothelial cells, and lymphocytes. While the role of Hla has been carefully elucidated in numerous animal models [14,15], most of the leukocidins exhibit a markedly increased tropism for human leukocytes in comparison to murine cells [6,16,17], likely resulting in a previous underappreciation of the importance of the leukocidins when extrapolating from murine data.

Since its discovery by two independent groups in 2010 [12,18], LukAB/LukGH has garnered attention as an important *S. aureus* virulence factor based on its clear role in both *ex vivo* and *in vivo* models of disease [6,12,13,19]. Infection of human neutrophils with diverse *S. aureus* strains indicates that LukAB/LukGH is the dominant toxin responsible for neutrophil targeting and killing [12]. This toxin is also highly conserved, being present in the genome of all known clinical isolates tested to date [20,21]. Finally, LukAB/LukGH is clearly produced during human infection, as evidenced by its recognition by the humoral response following invasive human disease [21,22].

In this issue of *Virulence*, Rouha *et al* have thoroughly evaluated the capacity of a pair of human monoclonal antibodies to inhibit the cytotoxicity of the leukocidins and Hla [23]. These antibodies, termed ASN-1 and ASN-2, were isolated by screening a human IgG1 antibody library using a yeast selection system; ASN-1 exhibits cross-reactive neutralizing activity against Hla and four of the leukocidins (PVL, LukED, and the γ-hemolysins), while ASN-2 neutralizes LukAB/LukGH. The authors have previously reported the cross-reactive capacity of ASN-1 [24], itself an important discovery given the redundant nature of *S. aureus* virulence factor expression. In this report, Rouha and colleagues characterize the individual and combined effects of the mAbs in a variety of *in vitro* models using human leukocytes, an important distinction given leukocidin tropism.

Several notable findings emerge from this work. First, the authors observed marked differences in toxin production in the presence of different culture media, particularly for the leukocidins. This speaks to the difficulty of interpreting the importance of staphylococcal toxins (and many other virulence factors) from different *in vitro* models, as protein expression by *S. aureus* may vary dramatically based on factors such as pH, oxygen tension, and nutrient availability [13,25,26]. Of note, the authors found that LukAB/LukGH was the dominant toxin in the media that may best recapitulate the host environment in the setting of human infection, RPMI + Casamino acids. Second, the authors observed that toxin production also varied widely across *S. aureus* strains. As the pore-forming toxins are evaluated as putative targets of intervention against *S. aureus*, it will be important to define the critical toxin(s) in the setting of human infection and produced by widely circulating clinical isolates. The authors also found that both antibodies were required to fully prevent toxin-mediated lysis of human neutrophils in most *in vitro* conditions, emphasizing the apparent redundancy in this pathway, though caution must be used when extrapolating these *in vitro* findings to what occurs in the human host during natural infection.

Many fascinating questions remain unanswered regarding pore-forming toxin biology and the interaction between these important *S. aureus* virulence factors and the human host. Despite the robust *in vitro* characterization of antibody-mediated pore-forming toxin inhibition reported by Rouha and colleagues[23], gaps remain in our understanding of antibody-toxin interactions in the setting of serious human infections, the setting in which a putative therapeutic would be deployed. For example, our group recently reported that different human antibodies (purified from B-cells obtained from children with invasive *S. aureus* disease) neutralize LukAB/LukGH-mediated cytotoxicity by distinct mechanisms [22]. It remains unclear whether certain of these mechanisms are more biologically relevant or important in the setting of invasive human infection. Further, evidence of antibody-enhanced disease has been reported in a murine model for at least one of the leukocidins (PVL) [27], and the relevance of this in humans (both for PVL and the other leukocidins) remains largely unexplored. The authors note that the antibody combination reported in this manuscript is under investigation in a Phase II clinical trial involving mechanically ventilated patients heavily colonized with *S. aureus*. The findings of this and other future work will hopefully provide further insights into the potential roles of antibody-mediated neutralization of pore-forming toxins in the setting of human disease.
